# Changes in intestinal microbiota across an altitudinal gradient in the lizard *Phrynocephalus vlangalii*

**DOI:** 10.1002/ece3.4029

**Published:** 2018-04-15

**Authors:** Wenya Zhang, Na Li, Xiaolong Tang, Naifa Liu, Wei Zhao

**Affiliations:** ^1^ Gansu Key Laboratory of Biomonitoring and Bioremediation for Environmental Pollution School of Life Sciences Lanzhou University Lanzhou China

**Keywords:** altitude, hypoxia, intestinal microbiota, *Phrynocephalus vlangalii*, Qinghai‐Tibet Plateau

## Abstract

High altitude is an important driving force in animal evolution. However, the effect of altitude on gut microbial communities in reptiles has not been examined in detail. Here, we investigated the intestinal microbiota of three populations of the lizard *Phrynocephalus vlangalii* living at different altitudes using 16S rRNA gene sequencing. Bacteroidetes, Firmicutes, and Proteobacteria were the most abundant phyla. *Bacteroides*,* Odoribacter*, and *Parabacteroides* were the most abundant genera. Significant differences in the intestinal microbiota composition were found among the three populations from different altitudes. The proportions of Verrucomicrobia and *Akkermansia* decreased, whereas *Bacteroides* increased significantly with altitude. Greater abundance of *Bacteroides* at higher altitude led to the fractional increase in the phylum Bacteroides relative to other phyla. Hypoxia may be the main factor that caused intestinal microbiota variation in *P. vlangalii* along the altitude gradient. Overall, our study suggested that the community composition and structure of intestinal microbiota of the lizard *P. vlangalii* varied along altitudes, and such differences likely play a certain role in highland adaptation. Our findings warrant a further study that would determine whether ambient and body temperatures play a key role in the modulation of intestinal microbiota in reptiles.

## 1 INTRODUCTION

Native highland species, especially ectotherms, withstand a strong selective pressure. The temperature drops roughly 0.6°C every 100 m above the sea level, and the air pressure also decreases with elevation. As body temperature of ectotherms is highly dependent on the ambient temperature (Bakken & Angilletta, 2014), the effective activity time of highland ectotherms is very limited. This circumstance affects energy intake and determines the evolution of most life history traits of highland ectotherms, such as body size, sexual mature size and age (Angilletta, Niewiarowski, Dunham, Leaché, & Porter, 2004; Li, Zhou, & Liu, 2014; Sears, 2005), reproductive effort, and offspring size (Jin, Li, & Liu, 2013; Jin & Liu, 2007; Li et al., 2014). In addition, hypoxia in highland species can lead to metabolic dysfunctions that further aggravate the above process.

One approach to overcome the negative influence of highland environment is to balance energy budget. On one hand, highland ectotherms cut down their energy expenditure. Ectotherms exposed to highland conditions (in their natural habitat or through hypoxia and low‐temperature treatment) generally possess a lower basal metabolic rate (Li, Liang, et al. 2016), a lower growth rate (Sears, 2005), and a decreased reproductive effort (Jin & Liu, 2007; Jin et al., 2013; Li et al., 2014) compared to those in individuals inhabiting lowlands. On the other hand, highland ectotherms also increase their energy production. At low temperature and under hypoxic conditions, highland ectotherms can provide a steady flow of energy for life by increasing their heart rate (He, Xiu, Tang, Wang, et al. 2013), blood oxygen affinity (He, Xiu, Tang, Yue, et al. 2013; Lu et al., 2015), lung and/or heart mass (Han et al., 2016; He, Xiu, Tang, Yue, et al. 2013) as well as by decreasing aerobic respiration (Tang et al., 2013).

Gut microbiota, which assists food digestion and absorption, plays a key role in the energy budget of its host (Pi, Gao, & Zhu, 2017; Tremaroli & Backhed, 2012). The intestinal tract mass of highland animals is always directly proportional to the altitude (Han et al., 2016), which suggests that digestive and absorptive functions are under selection. Studies that utilized acute hypoxic exposure indicated that air pressure is an important exogenous factor that strongly modulates the composition of intestinal microbiota (Maity, Adak, Ghosh, Pait, & Mondal, 2013; Maity, Adak, Pathak, Pati, & Mondal, 2012). Comparative studies in pikas and humans living at different altitudes also confirmed that gut microbiota composition is influenced by altitude (Li & Zhao, 2015; Li, Li et al., 2016; Li, Gesang, et al. 2016). However, the response of intestinal microbiota during highland adaptation in reptiles has been rarely reported.

**Figure 1:**
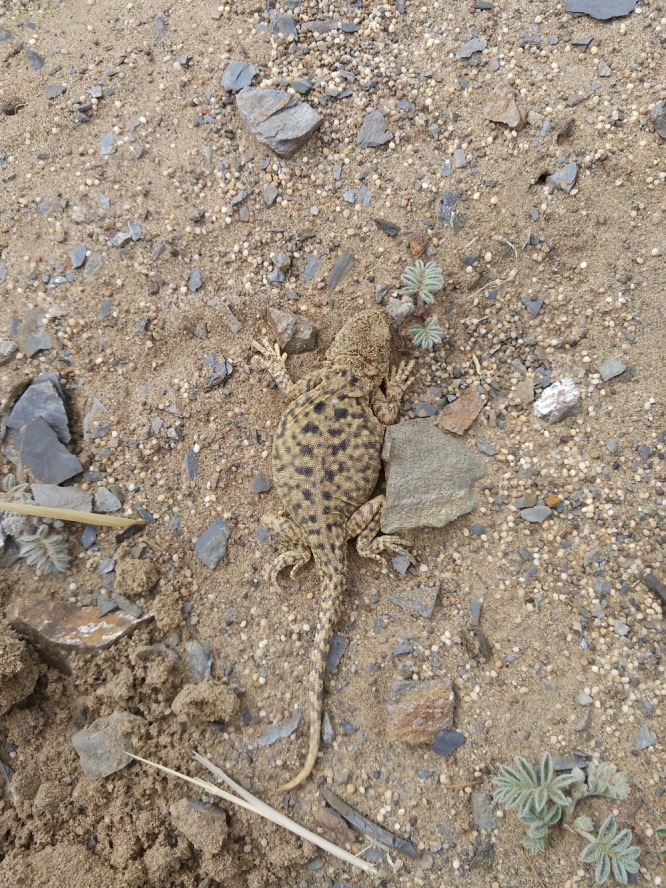
Photograph of female *Phrynocephalus vlangalii* from Delingha

**Figure 2:**
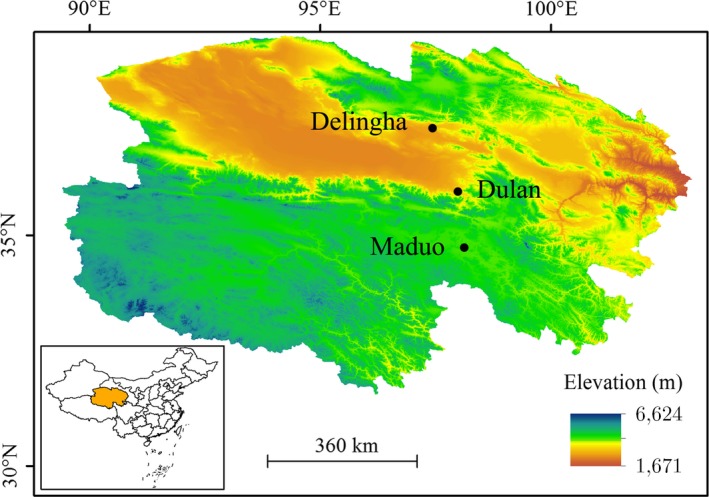
Sampling sites of *P. vlangalii*. DLH = Delingha (2,900 m ASL), DL = Dulan (3,338 m ASL), and MD = Maduo (4,250 m ASL). The elevation gradient is represented by graduated color, from yellow to blue (low to high). The map was created by ArcGIS 10.0 basing on the open data of Earth Resources Observatory and Science (EROS) Center (http://eros.usgs.gov/#)

The toad‐headed lizard *Phrynocephalus* *vlangalii* (Figure 1) is widely distributed on the Tibetan Plateau, where it inhabits a range of altitudes. *P. vlangalii* has been an ideal organism for evolutionary and ecological studies (Han et al., 2016; He, Xiu, Tang, Yue, et al. 2013; Jin & Liu, 2007; Jin et al., 2013; Li et al., 2014; Li, Liang, et al. 2016; Yang, Qi, & Fu, 2014). Through millions of years of evolution, *P. vlangalii* has evolved many highland‐adaptive traits in molecular biology (Yang et al., 2014), physiology (Han et al., 2016; He, Xiu, Tang, Yue, et al. 2013; Li, Liang, et al. 2016), and life history (Jin & Liu, 2007; Jin et al., 2013; Li et al., 2014). Because ecology and evolution of the host are tightly dependent on its gut microbiota composition (Colston & Jackson, 2016), we hypothesized that bacterial community composition and diversity in *P. vlangalii* intestines will vary with altitude. Using 16S rRNA gene sequencing technology, we analyzed intestinal microbiota composition of *P. vlangalii* along an altitude gradient (Figure 2).

## 2 MATERIALS AND METHODS

### 2.1 Study area and sampling

Fifteen adult *P. vlangalii* males of similar body size were captured randomly at three sites on the Qinghai‐Tibet Plateau along an altitude gradient in July 2015: DLH (N 37°9′19.60′′, E 97°35′27.07′′; 2,900 m), DL (N 36°22′3.03′′, E 98°12′22.90′′; 3,338 m), and MD (N 34°36′20.99′′, E 98°7′49.83′′; 4,250 m; Figure 2). Geographical location of sampled sites was determined using a Garmin Oregon E20 handheld GPS unit (Garmin, USA). A 10‐year cumulative climate data of each sample site were collected from the nearby climatic station (no more than 50 km away) of the Chinese National Climatic Data Center. To eliminate the difference between individuals caused by age, 13 samples of the same age (verified by skeletochronological analysis) were used for this study (Table 1). All the habitats belonged to desertified grassland covered with sparse vegetation, occasionally interspersed with few *Nitraria tangutorum* shrubs in DLH. Samples were stored in liquid nitrogen immediately after capture and transferred to the laboratory for further processing. Animal studies and experiments were approved by the Lanzhou University's Institutional Animal Care and Use Committee and carried out according to its guidelines.

**Table 1:** Sample information of each population

Population	Age	SVL (mm)	TL (mm)	BM (g)
DLH (*n* = 5)	3	56.17 ± 0.89	59.71 ± 4.23	6.84 ± 0.71
DL (*n* = 4)	3	56.62 ± 1.02	49.42 ± 2.31	5.97 ± 0.25
MD (*n* = 4)	3	54.80 ± 1.02	57.09 ± 1.77	6.52 ± 0.44

BM, body mass; SVL, snout‐vent length; TL, tail length. DLH = Delingha; DL = Dulan; MD = Maduo.

### 2.2 DNA extraction and amplification

Stomach was removed before procedure, and large contents in the intestine were carefully removed to avoid peeling off gut tissue. Afterward, the full intestinal tract was dissected and collected into tubes in sterile conditions. Total genomic DNA extraction was performed using a Fecal DNA Extraction Kit from Sangon (DP328, Sangon, Shanghai, China). Quantification of DNA was carried out using Qubit@ 2.0 Fluorometer (Thermo Scientific, USA). DNA purity was monitored on 1% agarose gels. DNA was diluted to 1 ng/μl using sterile water.

Primer 515F‐806R was used for 16S V4 (Caporaso et al., 2010). 16S rRNA genes were amplified using the specific primer with the barcode. All PCR reactions were carried out in a total volume of 30 μl reaction system, made up with 15 μl of Phusion^®^ High‐Fidelity PCR Master Mix (E0553L, Biolabs, New England), 0.2 μmol/L solutions of forward and reverse primers, and 10 ng of template DNA. Thermal cycling consisted of the initial denaturation at 98°C for 1 min, followed by 30 cycles of denaturation at 98°C for 10 s, annealing at 50°C for 30 s, and elongation at 72°C for 60 s. Finally, the PCR system was held at 72°C for 5 min. PCR products were detected by electrophoresis on a 2% agarose gel and the bands between 400–450 bp were purified with a GeneJET Gel Extraction Kit (Thermo Scientific, USA) for further experiments.

### 2.3 Library preparation and sequencing

Sequencing libraries were generated using a TruSeq^®^ DNA PCR‐Free Sample Preparation Kit (Illumina, USA) following manufacturer's recommendations and index codes were added. The library quality was assessed using Qubit@ 2.0 Fluorometer (Thermo Scientific, USA) and Agilent Bioanalyzer 2100 system. Finally, the library was sequenced on an Illumina HiSeq 2500 platform and 250 bp paired‐end reads were generated.

### 2.4 Sequence assembly and taxonomic identification

After trimming the barcode and primer sequence, paired‐end reads from the original DNA fragments were merged using FLASH (V1.2.7; Magoč & Salzberg, 2011), and the joined raw reads were analyzed using QIIME (V1.7.0; Caporaso et al., 2010). The chimera sequences were detected and removed using the UCHIME algorithm (Edgar, Haas, Clemente, Quince, & Knight, 2011).

Sequence analysis was performed by UPARSE software (UPARSE v7.0.1001; Edgar, 2013), and sequences with ≥97% similarity were assigned to the same operational taxonomic unit (OTU). Then, GreenGene database (GG 13.5) based on the RDP classifier algorithm (v2.2; Wang, Garrity, Tiedje, & Cole, 2007) was used to annotate taxonomic information. OTU abundance information was normalized using a standard sequence number corresponding to the sample with the least number of sequences. Subsequent analyses of alpha diversity and beta diversity were all performed based on this normalized data output.

### 2.5 Statistical analysis

To evaluate the complexity of intestinal microbiota, observed OTUs, alpha diversity (Shannon), richness (Chao), Good's coverage, and beta diversity were calculated with QIIME (v1.7.0) based on both weighted and unweighted UniFrac metrics and displayed with R software (v2.15.3).

To evaluate the differences in intestinal microbial composition and structure along an altitude gradient, the principal coordinate analysis (PCoA) was performed based on the weighted UniFrac metric by WGCNA package as well as by stats and ggplot2 packages in R software. To identify differences in microbial communities between the two groups, analysis of similarities (ANOSIM; Clarke, 1993) was performed based on the Bray–Curtis dissimilarity distance matrices by vegan package in R software. The linear discriminant analysis (LDA), as implemented in LEfSe software (Segata et al., 2011), was used to search for the taxon for which the relative abundance was significantly different among the various populations. In addition, the redundancy analysis (RDA) of principal coordinates was used to evaluate the significance of altitude, temperature, or hypoxia using vegan package in R software.

The variations in microbial count, alpha index, and beta index were examined by the Kruskal–Wallis one‐way analysis of variance. The Spearman's rank correlation test was used to determine whether the relative abundance of intestinal microbiota was significantly related to environmental factors (temperature and air pressure). Statistical analysis was conducted with SPSS 19.0 for Windows. Values are expressed as the means ± standard deviation (SD). All statistical analyses were conducted with a significance level of α = 0.05 (*p *<* *.05).

## 3 RESULTS

In total, 687,335 high‐quality (>Q30) reads were filtered from 713,928 raw reads obtained from 13 intestinal content samples. The total number of OTUs at the 97% similarity level was 9,025 (Table 2). The Shannon–Wiener curve of all samples suggested that enough OTUs have been detected as the rarefaction has levelled out, and thus did not limit their interpretation (Figure S1). Estimators of the Good's coverage, community richness (Chao), and diversity (Shannon index) are summarized in Table 2.

**Table 2:** Summary of Illumina HiSeq data of *P. vlangalii* microbiota

Population	Elevation (m ASL)	Temperature (°C)	Air pressure (hPa)	Total reads	Taxon reads	OTUs	Shannon	Chao	Good's coverage
DLH (*n* = 5)	2,900	4.4	708.7	54,723 ± 6,578	52,823 ± 6,386	737.2 ± 135.9	5.929 ± 0.231	747.7 ± 130.9	0.995 ± 0.001
DL (*n* = 4)	3,338	3.4	691.3	51,139 ± 2,570	49,357 ± 2,726	723.8 ± 81.2	6.159 ± 0.084	752.9 ± 60.6	0.995 ± 0.001
MD (*n* = 4)	4,250	−3.3	604.3	58,940 ± 2,717	56,449 ± 2,585	611.0 ± 217.0	4.191 ± 0.982	667.4 ± 257.0	0.995 ± 0.003
Total				713,928	687,335	9,025			

All indices were calculated at the 97% similarity level. The number in the parentheses represents the sample size. DLH = Delingha, DL = Dulan, MD = Maduo.

### 3.1 Intestinal microbiota composition

**Figure 3:**
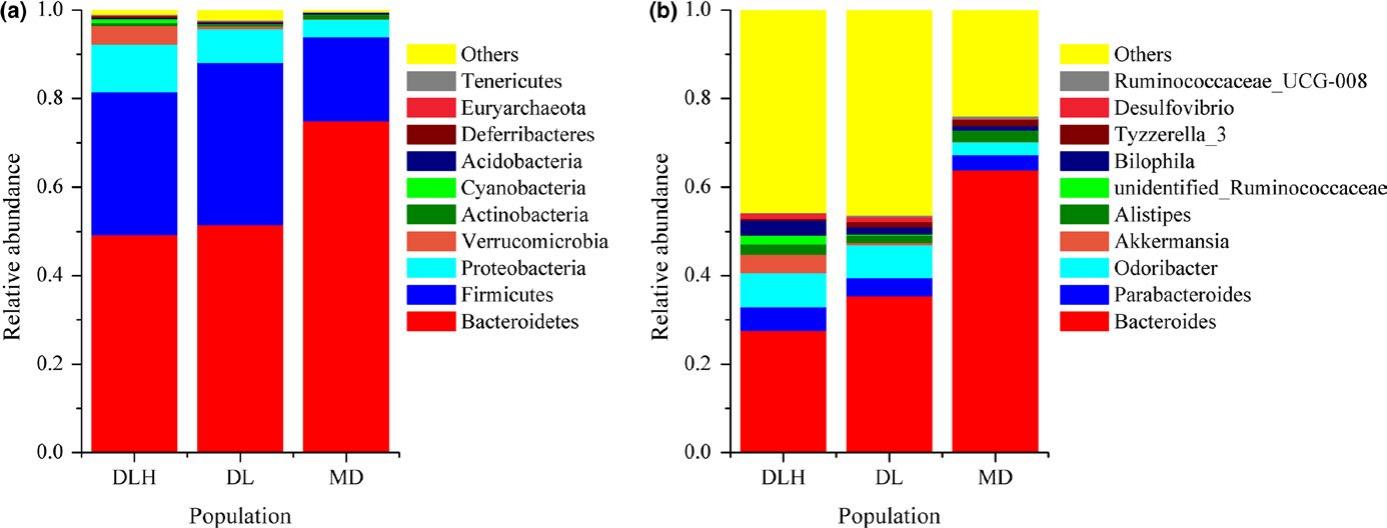
Composition of intestinal microbiota at the phylum (a) and genus (b) levels. DLH = Delingha, DL = Dulan, and MD = Maduo. Only the top ten phyla or genera are shown in histogram and the other taxons are combined

Overall, representatives of 29 phyla and 373 genera were found in intestinal samples of *P. vlangalii*. The composition of intestinal microbiota varied among the three populations. In similarity with the data from other vertebrates, the dominant phyla in all populations were Bacteroidetes, Firmicutes, and Proteobacteria (Figure 3a). Interestingly, there was no significant difference in the relative abundance of these three main microbial phyla (Kruskal–Wallis ANOVA, *p *>* *.051 in all cases), despite Bacteroidetes exhibited a tendency to be more predominant at higher altitude, whereas Firmicutes and Proteobacteria demonstrated an opposite trend (Figure 3a; Table S1). Notably, the abundance of Verrucomicrobia and Deferribacteres, which represented a very small proportion of microbiota (1.84% and 0.19% on average across all samples, respectively), decreased drastically with altitude (*p *=* *.009 and .022, respectively; Figure S2).

At the genus level, *Bacteroides* was the predominant bacterial genus. Its relative abundance varied significantly among populations at different altitudes (*p *=* *.012, Table S1) and comprised 27.63%, 35.42%, and 63.83% in Delingha (DLH), Dulan (DL), and Maduo (MD), respectively. The other nine main genera, which comprised more than 0.5% of the total genus composition each, were *Odoribacter*,* Parabacteroides*,* Alistipes*,* Bilophila*,* Akkermansia*,* Tyzzerella_3*,* Desulfovibrio*,* Anaerotruncus*, and *Hungatella* (Figure 3b; Table S1). Among these nine genera, only relative abundance of *Akkermansia* was significantly affected by altitude (*p *=* *.006, Table S1). In other rare (<0.5%) genera, only *Oscillospira*,* Mucispirillum*, and *Intestinimonas* varied significantly among *P. vlangalii* populations at different altitudes (*p *=* *.047, .022, and .038, respectively; Table S1). The relative abundances of these genera were inversely proportional to altitude (Table S1; Figure S3).

**Figure 4:**
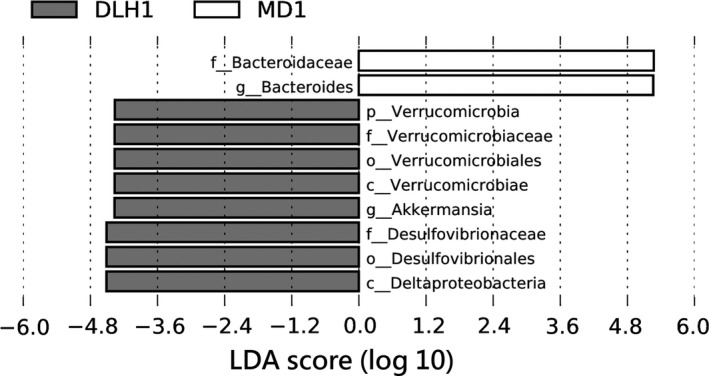
Differential abundance of several taxa in intestinal microbiota of the lizard *Phrynocephalus vlangalii* at different altitudes analyzed using LEfSe software (linear discriminant analysis [LDA]). Taxa enriched in high‐altitude MD population (4,250 m above the sea level) are indicated with a positive LDA score (white), whereas taxa enriched in low‐altitude DLH population (2,900 m) have a negative score (gray). Only taxa with an LDA exceeding the significant threshold of 2 are shown. DLH = Delingha, MD = Maduo

The taxonomic abundances in intestinal microbiota in *P. vlangalii* populations at the three altitudes were compared by the LEfSe analysis. A greater proportion of the family Bacteroidaceae and genus *Bacteroides* was found in high‐altitude population (MD) compared to their fraction in low‐altitude population (DLH). In contrast, the proportions of the phylum Verrucomicrobia, family Verrucomicrobiaceae, and genus *Akkermansia* in intestinal microbiota of low‐altitude *P. vlangalii* were greater than those in lizards at high altitude. The LEfSe analysis also demonstrated that the proportion of the family Deferribacteraceae in low‐altitude population was greater than that in high‐altitude population (Figure 4).

### 3.2 Diversity of intestinal microbiota in lizards at different altitudes

**Figure 5:**
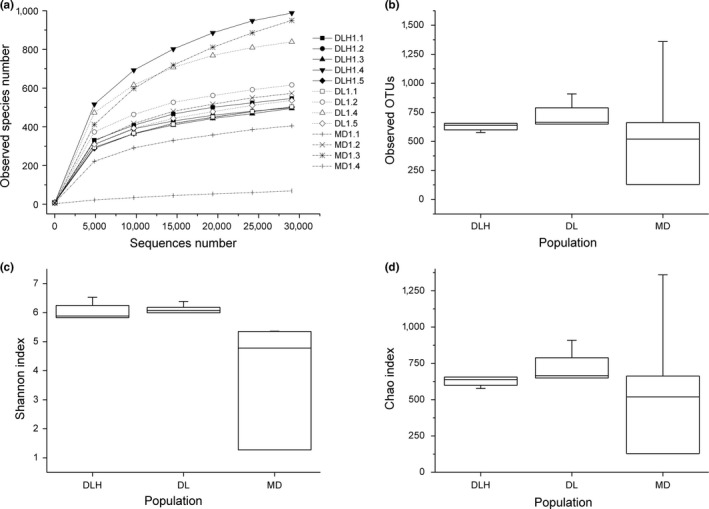
The rarefaction curves and alpha diversity indices of intestinal microbiota of the lizard *Phrynocephalus vlangalii* at different altitudes. (A) The rarefaction curves of the observed species, the solid line represents DLH, the dot line represents DL, and the dash line represents MD; (B) species observed; (C) Shannon index; (D) Chao index. DLH = Delingha, DL = Dulan, and MD = Maduo

Alpha diversity of intestinal microbiota was assessed by the observed species, Shannon, and Chao indices. OTU‐level rarefaction curves of the observed species indices across all samples reached stable values (Figure 5a), indicating that the sequencing was deep enough. There was no significant effect of altitude on the number of species observed (*p *=* *.634; Figure 5b) or the Chao index (*p *=* *.654, Figure 5d). Meanwhile, the Shannon index (community diversity) varied significantly among *P. vlangalii* populations at different altitudes (*p *=* *.038) with MD population having the lowest diversity (Figure 5c).

**Figure 6:**
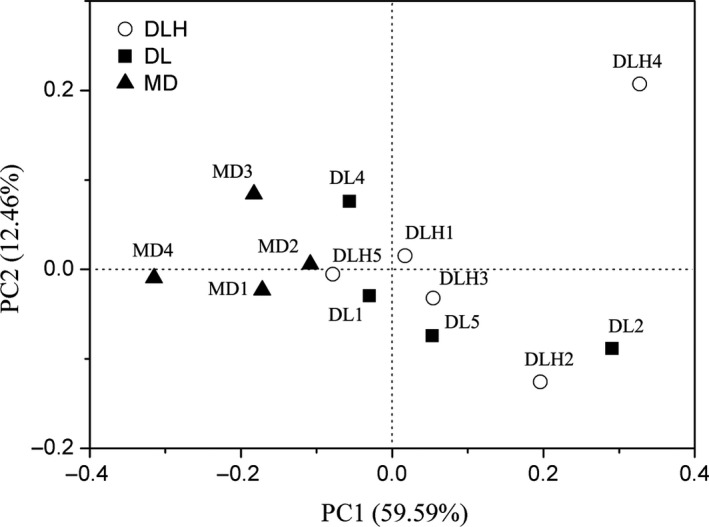
Principal coordinates analysis (PCoA) based on the weighted UniFrac metric of intestinal microbiota of the lizard *Phrynocephalus vlangalii* at different altitudes. The model gives three principal components and only the first two PCs are shown in the figure. The white circles represent samples from DLH, the black boxes represent samples from DL, and the black triangles represent samples from MD

For beta diversity analysis, we performed weighted UniFrac PCoA and found that there was an obvious separation of MD samples from other two groups. Pco1 and Pco2 accounted for 59.59% and 12.46% of the total variation, respectively (Figure 6). Analysis of similarities showed that intestinal microbiota was significantly different in lizards from DLH and MD locations (*r* = .375, *p *=* *.023), but was similar both between DLH and DL (*r* = .043, *p *=* *.326) and between DL and MD (*r* = .239, *p *=* *.057).

### 3.3 Correlation between intestinal microbiota and environmental factors

**Figure 7:**
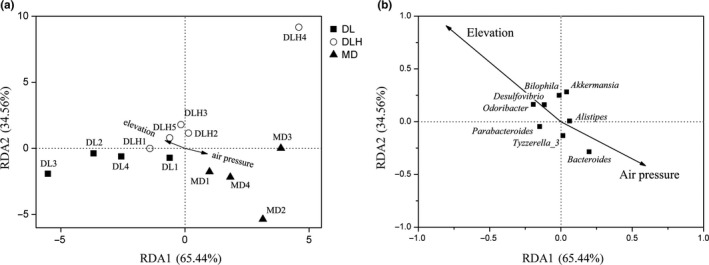
Biplot of redundancy analysis (RDA) of intestinal microbiota samples from the lizard *Phrynocephalus vlangalii* responding to environmental factors. As temperature and air pressure were significantly autocorrelated, the model retained just air pressure and elevation. The white circles represent samples from DLH, the black boxes represent samples from DL, and the black triangles represent samples from MD (a). The black boxes represent the intestinal microbiota at genus level and only those genera with relative abundance greater than 0.5% were shown (b)

Redundancy analysis (RDA) showed that intestinal microbiota of MD population of *P. vlangalii* was mainly affected by air pressure, whereas DLH population of lizards was mainly influenced by elevation (Figure 7a). RDA also showed that some bacterial genera were associated with certain environmental factors, such as *Bacteroides* significantly correlated with elevation and *Oscillospira* significantly correlated with air pressure (Figure 7b). The Spearman's rank correlation analysis indicated that the relative abundances of several intestinal microbiota genera correlated with environmental factors (Figure S4). The relative abundance of *Bacteroides* positively correlated with elevation but negatively correlated with temperature and air pressure (*p *<* *.001 in all cases). The relative abundances of *Akkermansia*,* Mucispirillum*,* Bilophila*,* Ruminococcaceae_UCG.014*,* Tyzzerella*,* Oscillospira*, and *Intestinimonas* positively correlated with temperature and air pressure but negatively correlated with elevation (*p *<* *.05 in all cases).

## 4 DISCUSSION

The complex and diverse microbiota in the gastrointestinal tract plays an important role in maintaining host biological functions, including nutrient metabolism (Sekirov, Russell, Antunes, & Finlay, 2010; Tremaroli & Backhed, 2012). A growing number of studies have indicated that animals inhabiting high‐altitude environments possess distinct gastrointestinal microbiota compared to that of representatives of the same species that live at low altitude (Li & Zhao, 2015; Li, Li et al., 2016; Li, Gesang, et al. 2016). In the present study, we also found that *P. vlangalii* lizards inhabiting different altitudes have relatively different intestinal microbiota and confirmed that geography and climate are significant factors regulating gut microbiota of the reptiles on the Tibetan Plateau.

Similar to the results of other studies in reptiles (Colston, Noonan, & Jackson, 2015; Kohl et al., 2016), we found that Bacteroidetes and Firmicutes were dominant intestinal microbiota species of *P. vlangalii*. Notably, the proportion of Bacteroidetes was higher than that of Firmicutes (Figure S1; *Z* = −2.551, *p *=* *.011, Wilcoxon rank test,) in *P. vlangalii*, which was a different pattern from that reported in previous studies of herbivorous lizards (Hong, Wheeler, Cann, & Mackie, 2011; Kohl et al., 2016). As diet plays a key role in determining the composition of gastrointestinal microbial communities, this difference may be explained by the fact that *P. vlangalii* is a carnivore (Bao, Zeng, Ma, & Yuan, 1998). Considering that gut *Bacteroides* spp. also contribute to the degradation of ingested heterologous polysaccharides in plant material (Patrick, 2015), this difference may also depend on some other unknown factors, such as host phylogeny or geographical location (Lankau, Hong, & Mackie, 2012).

The relative abundance of the phyla Bacteroidetes in the intestines of *P. vlangalii* lizards tended to be higher with altitude, which was mainly caused by a significantly increasing fraction of the genus *Bacteroides*. In contrast, the relative abundance of the phyla Firmicutes was inversely proportional to the altitude. Our results are distinctly different from the observations in humans (Li & Zhao, 2015; Li, Gesang, et al. 2016), but are similar to the findings in pikas (Li et al., 2016a). Despite observations in these different species are dissimilar, it is possible that the Firmicutes/Bacteroidetes (F/B) ratio is determined by the diet. For example, the F/B ratio, which has been used as specific index of gut microbiota, is affected by the caloric value of the diet (Murphy et al., 2010). Different F/B ratios in Tibetans and Chinese Han may be explained by distinct dietary habits (Li & Zhao, 2015). Whereas the increase in the proportion of Bacteroidetes and relative decrease in Firmicutes may help pikas to digest plant cellulose more efficiently (Li, Li, et al. 2016), *P. vlangalii* seems to consume exclusively insects (Bao et al., 1998), and the information about their diet variation is lacking. In addition, soil bacteria and the microbes injected with invertebrate diet have been proved to contribute little to the gut microbiota of lizards (Kohl et al., 2016). Therefore, whether the lower F/B ratio at high altitude, caused by the higher proportion of Bacteroidetes and lower fraction of Firmicutes, was induced by diet variation or by other unknown factors requires a further study.

As it had been observed in other reptiles (Colston & Jackson, 2016; Kohl et al., 2016), Proteobacteria was the third most abundant phylum in the intestinal microbiota of *P. vlangalii*. In addition, we also found a relatively high proportion of the phylum Verrucomicrobia, which was the fourth most abundant phylum in the intestinal microbiota of *P. vlangalii*. The proportions of both these phyla tended to become lower with the increase in altitude, and the variation in Verrucomicrobia fraction among the three sites was statistically significant. The decrease in Verrucomicrobia abundance with altitude was mainly due to a significant decrease in the genus *Akkermansia*. Proteobacteria are metabolically diverse and typically break down and ferment complex sugars as well as produce vitamins (Colston & Jackson, 2016). In addition, many studies indicated that the relative abundance of *Akkermansia* increases under caloric restriction conditions (Derrien, Belzer, & de Vos, 2016). These variations may imply *P. vlangalii* living at high altitude had abundant food supply. Actually, studies of insect richness on the Qinghai‐Tibet Plateau demonstrated that the numbers of Hymenoptera insects increase as altitude goes up (Liu, Gong, Shi, Yang, & Feng, 2007).

Notably, the microbial community structure and species richness of intestinal microbiota were also affected by altitude gradient. Both Kruskal–Wallis ANOVA and LEfSe analysis indicated that almost all significant variations in proportions were found between the lowest and highest populations of *P. vlangalii* (Figure 5; Table S1). PCoA also suggested that the intestinal microbiota community structure was not different between DLH (2,900 m) and DL (3,338 m) sites, or between DL (3,338 m) and MD (4,250 m) sites. These findings indicated that a small altitude gradient may not be sufficient to cause a significant variation in *P. vlangalii* intestinal microbiota. Nonetheless, it should be noted that studies in pika found a significant variation in gut microbiota along a relative small altitude gradient (between 3,694 m and 4,331 m; Li, Li, et al. 2016). It is slightly counterintuitive, because considering that *P. vlangalii* is an ectothermic species, its intestinal microbiota should be more sensitive to the environmental variation than that of pika. Thus, further studies are needed to examine such potentially higher sensitivity.

Hyperbaric pressure, which is a typical characteristic of high altitude, has been proved as an important factor that strongly modulates the composition of gut microflora in mammals (Adak, Maity, Ghosh, Pati, & Mondal, 2013; Li, Li, et al. 2016; Maity et al., 2012, 2013). Our results also confirmed that hypoxia may affect intestinal microbial composition in *P. vlangalii*. Hypoxia may be the main factor that caused the increase in the fraction of *Bacteroides* and decrease in the proportion of *Akkermansia* along the altitude gradient. Low temperature, which significantly and positively correlates with air pressure, is another typical feature of high altitude. It has been proposed that water temperature may influence gut microbiota of fishes (Neuman et al., 2016). Despite body temperature of lizards highly depends on the ambient temperature, they can maintain a relatively stable body temperature through behavioral thermoregulation (Gvoždík & Castilla, 2001; Méndez‐Galeano & Calderón‐Espinosa, 2016). Therefore, the effect of low ambient temperature on intestinal microbiota may be buffered by thermoregulation, and our present results may reflect the effect of hypoxia. A further study that would specifically examine the variation in body temperature of *P. vlangalii* living at different altitudes will contribute to our understanding of this issue.

In conclusion, our study revealed that the community composition and structure of intestinal microbiota of the lizard *P. vlangalii* varied at different altitudes, and such differences likely play a certain role in highland adaptation. These variations in intestinal microbiota may be mainly attributed to the effect of hypoxia and other unknown factors. Further controlled animal experiments are needed to elucidate whether preferred body temperatures of *P. vlangalii* or other potentially important environmental variables like ambient temperature play a key role in regulating intestinal microbiota. The functional significance of these changes in intestinal microbiota during high‐altitude adaption for reptiles also remains to be established.

## AUTHOR CONTRIBUTIONS

Z.W. and N.F.L. designed this experiment. Z.W., L.N., and T.X.L. collected the samples. Z.W.Y. and L.N. performed the experiment. Z.W. analyzed the data. Z.W. and Z.W.Y. drafted the manuscript.

## CONFLICT OF INTEREST

None declared.
