# Supplementary figures and images for: Changes in intestinal microbiota across an altitudinal gradient in the lizard Phrynocephalus vlangalii
Source: Ecol Evol. 2018 Apr 15;8(9):4695–703. doi: 10.1002/ece3.4029 (PMC5938461; doi:10.1002/ece3.4029)

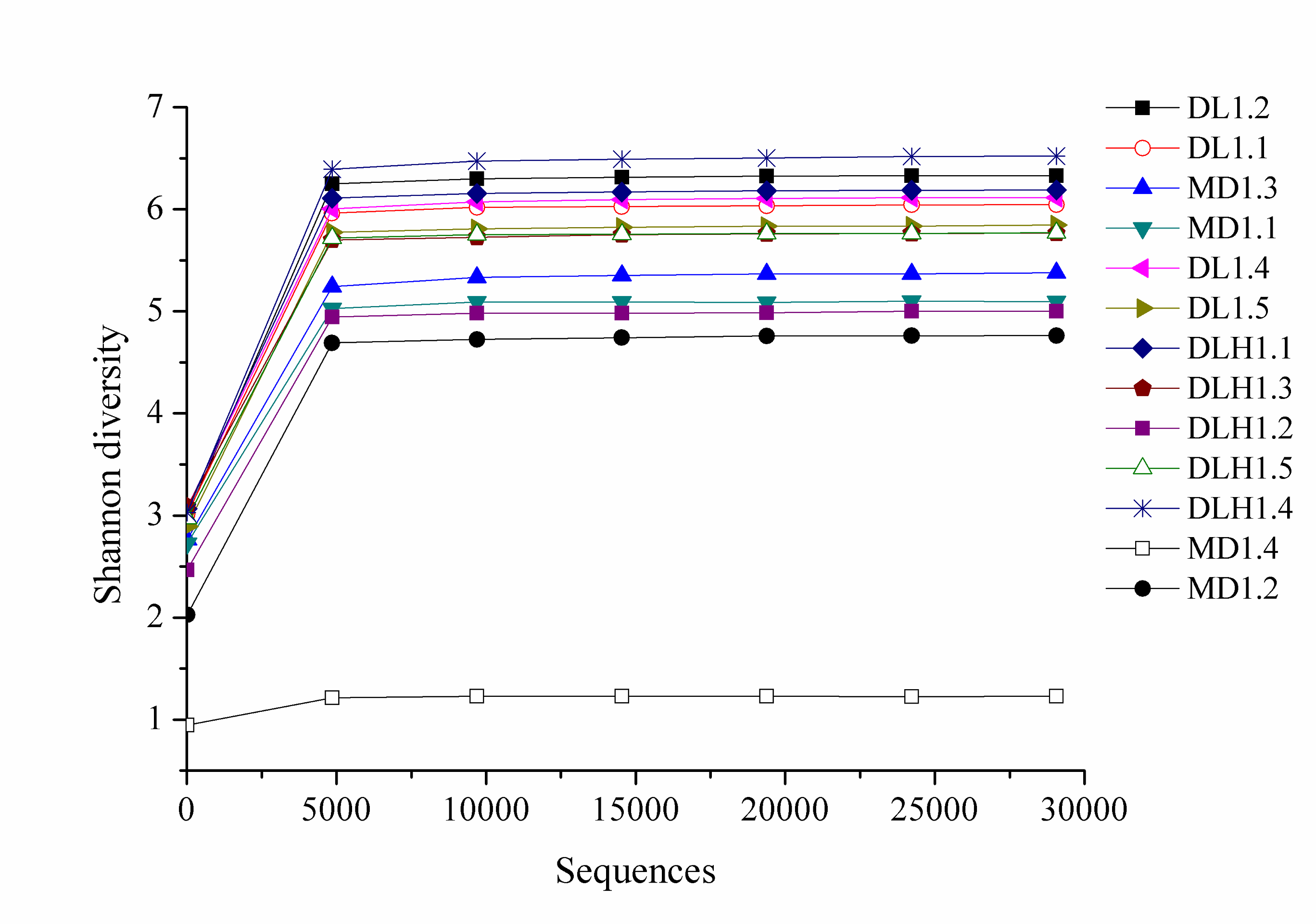

Supplement: Supplementary file 1 [file ECE3-8-4695-s001.tif]
