# Supplementary material for: Changes in intestinal microbiota across an altitudinal gradient in the lizard Phrynocephalus vlangalii
Source: Ecol Evol. 2018 Apr 15;8(9):4695–703. doi: 10.1002/ece3.4029 (PMC5938461; doi:10.1002/ece3.4029)

**Changes in intestinal microbiota reflecting highland adaptation in the lizard *Phrynocephalus vlangalii***

Wenya Zhang, Na Li, Xiaolong Tang, Naifa Liu, Wei Zhao*

**Supplementary Information**

Supplementary Table. S1. The microbial taxa (mean relative abundance > 0.1%) of *Phrynocephalus vlangalii* intestinal microbiota at phylum and genus level across different populations.

| phyla (>0.1%) | DLH | DL | MD | P |
| --- | --- | --- | --- | --- |
| Bacteroidetes | 49.26 ± 16.58 | 51.54 ± 14.99 | 74.99 ± 9.73 | 0.066 |
| Firmicutes | 32.31 ± 11.88 | 36.61 ± 15.31 | 19.00 ± 6.51 | 0.086 |
| Proteobacteria | 10.68 ± 9.79 | 7.72 ± 4.79 | 3.88 ± 2.85 | 0.234 |
| Verrucomicrobia | 4.23 ± 3.92a | 0.51 ± 0.33ab | 0.18 ± 0.12b | 0.009 |
| Actinobacteria | 0.74 ± 0.81 | 0.42 ± 0.55 | 0.95 ± 1.21 | 0.570 |
| Cyanobacteria | 0.87 ± 0.77 | 0.17 ± 0.08 | 0.13 ± 0.11 | 0.051 |
| Acidobacteria | 0.25 ± 0.54 | 0.34 ± 0.67 | 0.34 ± 0.65 | 0.757 |
| Tenericutes | 0.29 ± 0.14 | 0.23 ± 0.08 | 0.15 ± 0.13 | 0.263 |
| Deferribacteres | 0.38 ± 0.52a | 0.11 ± 0.06ab | 0.03 ± 0.04b | 0.022 |
|  |  |  |  |  |
| genera (>0.1%) |  |  |  |  |
| *Bacteroides* | 27.63 ± 9.44a | 35.42 ± 13.98ab | 63.83 ± 14.70b | 0.012 |
| *Odoribacter* | 7.75 ± 3.48 | 7.46 ± 3.42 | 2.84 ± 3.28 | 0.134 |
| *Parabacteroides* | 5.31 ± 4.35 | 4.12 ± 1.86 | 3.52 ± 3.75 | 0.897 |
| *Alistipes* | 2.29 ± 2.02 | 1.68 ± 0.75 | 2.51 ± 4.38 | 0.376 |
| *Bilophila* | 3.41 ± 2.39 | 1.71 ± 0.96 | 0.99 ± 1.07 | 0.118 |
| *Akkermansia* | 4.19 ± 3.95a | 0.48 ± 0.34ab | 0.11 ± 0.12b | 0.006 |
| *Tyzzerella_3* | 0.35 ± 0.16 | 1.19 ± 1.08 | 1.42 ± 2.42 | 0.133 |
| *Desulfovibrio* | 1.17 ± 1.20 | 1.05 ± 1.03 | 0.18 ± 0.16 | 0.166 |
| *Anaerotruncus* | 0.66 ± 0.15 | 1.01 ± 0.42 | 0.33 ± 0.30 | 0.059 |
| *Hungatella* | 0.77 ± 0.50 | 0.64 ± 0.27 | 0.56 ± 0.56 | 0.710 |
| *Ruminiclostridium_5* | 0.34 ± 0.16 | 0.43 ± 0.21 | 0.38 ± 0.5 | 0.838 |
| *Eubacterium_coprostanoligenes_group* | 0.25 ± 0.12 | 0.59 ± 0.69 | 0.31 ± 0.33 | 0.647 |
| *Ruminococcaceae_UCG-008* | 0.19 ± 0.06 | 0.36 ± 0.20 | 0.60 ± 0.92 | 0.291 |
| *Thalassospira* | 0.22 ± 0.18 | 0.80 ± 0.75 | 0.11 ± 0.14 | 0.192 |
| *Candidatus_Captivus* | 0.36 ± 0.26 | 0.35 ± 0.41 | 0.24 ± 0.36 | 0.422 |
| *Ruminococcaceae_UCG-014* | 0.41 ± 0.24 | 0.34 ± 0.23 | 0.12 ± 0.09 | 0.054 |
| *Rikenella* | 0.26 ± 0.25 | 0.39 ± 0.14 | 0.24 ± 0.28 | 0.376 |
| *Candidatus_Hepatincola* | 0.56 ± 0.57 | 0.10 ± 0.03 | 0.07 ± 0.10 | 0.332 |
| *Oscillospira* | 0.27 ± 0.18a | 0.25 ± 0.10ab | 0.07 ± 0.05a | 0.047 |
| *Tyzzerella* | 0.20 ± 0.07 | 0.31 ± 0.27 | 0.08 ± 0.05 | 0.057 |
| *Mucispirillum* | 0.38 ± 0.52a | 0.11 ± 0.06ab | 0.03 ± 0.04b | 0.022 |
| *Intestinimonas* | 0.24 ± 0.17a | 0.26 ± 0.03ab | 0.05 ± 0.04b | 0.038 |
| *Lachnoclostridium* | 0.26 ± 0.21 | 0.16 ± 0.05 | 0.13 ± 0.12 | 0.548 |
| *Aeromonas* | 0.10 ± 0.06 | 0.18 ± 0.05 | 0.07 ± 0.11 | 0.217 |
| *Eisenbergiella* | 0.05 ± 0.02 | 0.10 ± 0.09 | 0.18 ± 0.29 | 0.694 |

Values labeled with different letters in a row mean significant difference at p=0.05(Kruskal-Wallis One-way ANOVA)

Supplementary Fig. S2. Heatmap of relative abundances of the phyla in intestinal microbiota of the lizard *Phrynocephalus vlangalii* at different altitudes. DLH = Delingha, DL = Dulan, MD = Maduo. The relative abundance is represented by graduated colour, from blue to red (low to high).


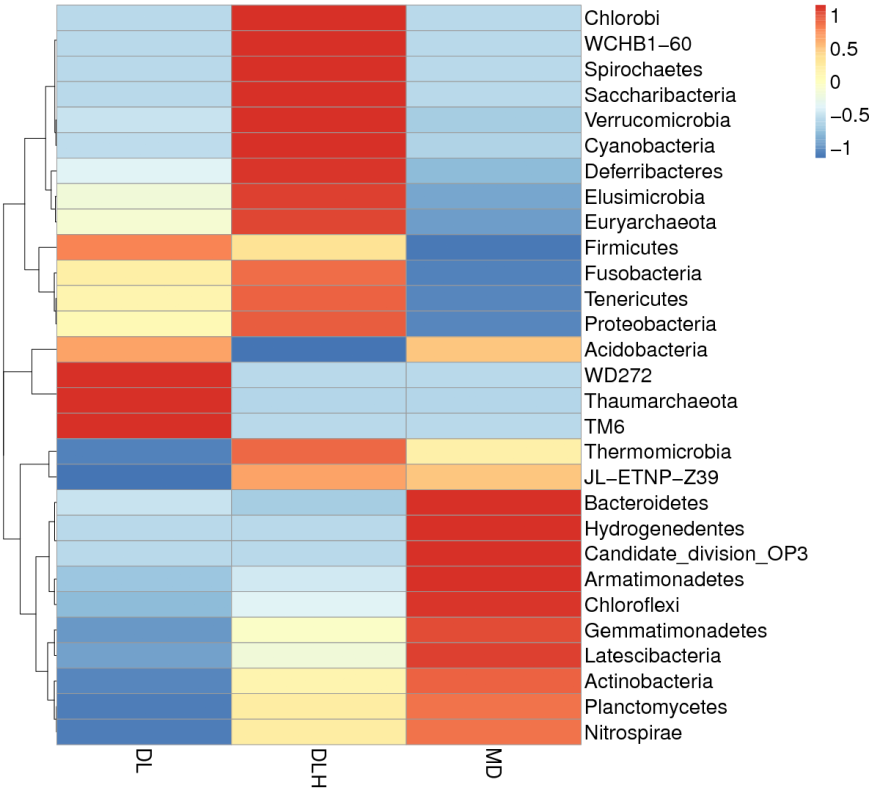


Supplementary Fig. S3. Heatmap of relative abundances of the genera in intestinal microbiota of the lizard *Phrynocephalus vlangalii* at different altitudes. DLH = Delingha, DL = Dulan, MD = Maduo. The relative abundance is represented by graduated colour, from blue to red (low to high).


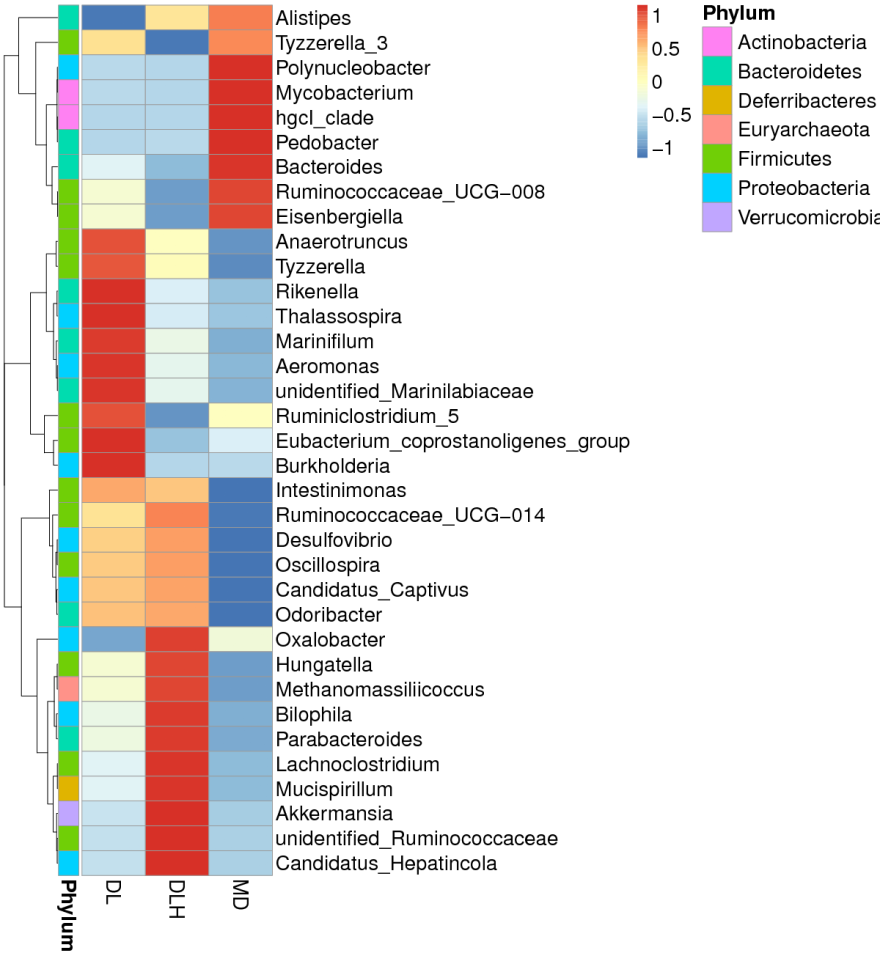


Supplementary Fig. S4. Spearman’s rank correlation between the relative abundances of intestinal microbiota of the lizard *Phrynocephalus vlangalii* and environmental factors (temperature, elevation, and air pressure. Positive correlation is shown in red and negative correlation — in blue. Statistical significance is indicated as **P* < 0.05 and ***P* < 0.01.


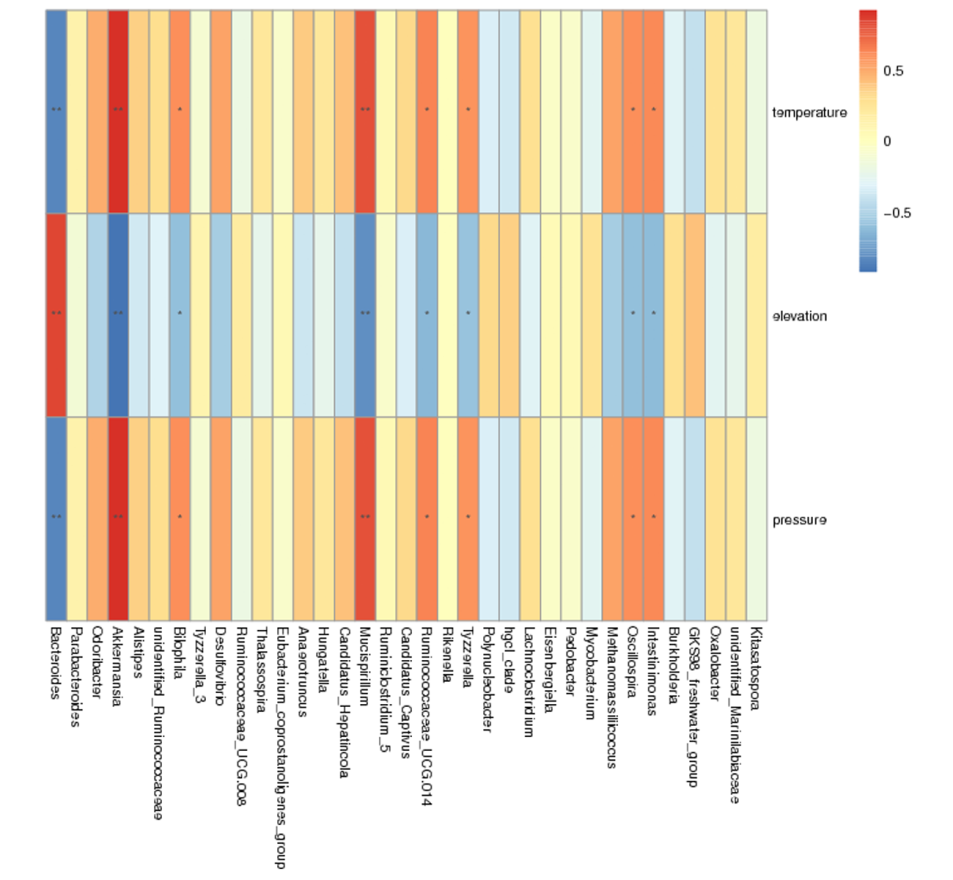

Supplement: Supplementary file 2 [file ECE3-8-4695-s002.doc]
